# Probabilistic Analysis of a Buffer Overflow Duration in Data Transmission in Wireless Sensor Networks

**DOI:** 10.3390/s20205772

**Published:** 2020-10-12

**Authors:** Wojciech M. Kempa

**Affiliations:** Department of Mathematics Applications and Methods for Artificial Intelligence, Faculty of Applied Mathematics, Silesian University of Technology, 44-100 Gliwice, Poland; wojciech.kempa@polsl.pl; Tel.: +48-32-237-2864

**Keywords:** buffer overflow, discrete time, packet loss, Quality of Service (QoS), wireless sensor network (WSN)

## Abstract

One of the most important problems of data transmission in packet networks, in particular in wireless sensor networks, are periodic overflows of buffers accumulating packets directed to a given node. In the case of a buffer overflow, all new incoming packets are lost until the overflow condition terminates. From the point of view of network optimization, it is very important to know the probabilistic nature of this phenomenon, including the probability distribution of the duration of the buffer overflow period. In this article, a mathematical model of the node of a wireless sensor network with discrete time parameter is proposed. The model is governed by a finite-buffer discrete-time queueing system with geometrically distributed interarrival times and general distribution of processing times. A system of equations for the tail cumulative distribution function of the first buffer overflow period duration conditioned by the initial state of the accumulating buffer is derived. The solution of the corresponding system written for probability generating functions is found using the analytical approach based on the idea of embedded Markov chain and linear algebra. Corresponding result for next buffer overflow periods is obtained as well. Numerical study illustrating theoretical results is attached.

## 1. Introduction

Capacities of buffers accumulating incoming packets in computer and telecommunication network nodes, e.g., wireless sensor network (WSN) nodes or LAN routers, are limited. As a consequence, one of typical phenomena of packet processing by a network node, especially when the traffic is heavy, is buffer overflows resulting in packet losses. As long as the accumulating buffer is overflowing, all incoming packets are lost. This causes deterioration of key service parameters, like packet loss ratio, end-to-end delay and mean energy consumption [1,2]. Obviously, as one can say, when a network sensor is turned on and the traffic is small, the problem of energy saving appears since we have long idle times. From the other side, sensor nodes are equipped typically with very small buffers, so during a “catastrophic” period, when large data amount arrives at the sensor node, buffer overflows and packet losses occur. Hence [3], reducing the power consumption during the “normal” period and reducing buffer overflow durations during the “catastrophic” period are equally important. In single-hop wireless sensor networks the sensor measurement is sent directly to the base station (sink node). However, in multi-hop topology the information is transferred from sensor to sensor to the sink node collecting the traffic, using a multi-hop protocol [2]. Unexpected buffer overflows occurring on the route (at intermediate nodes) generate packet losses and significantly reduce the Quality of Service (QoS). In fact, in the literature, one can find many available schemes of the buffer management dedicated to traditional wireless networks. However [4], they cannot be directly used in wireless sensor networks due to strong limitations in power supply and the memory.

The problems and challenges described above are the motivation for the in-depth analytical study of the buffer overflow phenomenon and its probabilistic nature. In particular, the analysis of the probability distribution of a single buffer overflow duration as a function of the buffer capacity, the intensity of packet arrivals and the processing speed is desirable.

In this paper, we consider a mathematical model of the node of a wireless sensor network with discrete time parameter. The model is based on a finite-buffer discrete-time queueing system with one processing station. As it seems, queueing systems with finite capacities of waiting rooms accumulating the incoming packets (customers, cells, jobs, etc.) have far greater potential practical applications compared to systems with infinite ones. Indeed, the enqueueing process of data packets arriving at nodes of computer or telecommunication networks (like IP routers or nodes of WSNs) or jobs occurring in the production process assumes a finite buffer (magazine) capacities. In consequence, a natural phenomena of a buffer overflow may appear, during which the “qualification” of new customers for the processing is timely suspended due to no place for waiting. Since during the buffer overflow all the entering customers are usually rejected without service, the knowledge of the probabilistic distribution of the duration of successive buffer overflow periods is essential from the point of view of the system control and optimization in order to ensure a proper level of the Quality of Service.

One can find analytical results for probability distributions of buffer overflow durations for the finite- and infinite-buffer M/G/1-type queueing models in [5,6,7,8]. In [9], an M/G/1/N-type system with a single vacation mechanism is analyzed. A compact-form representation for the Laplace transform (LT for short) of the first buffer overflow duration cumulative distribution function (CDF) is found there. Hence, the formula for the LT of next such periods is derived. The generalization of these results for the case of a multiple vacation policy and for the compound Poisson arrival stream can be found in [10,11], respectively [12,13]. Analytical results for the CDF of the time to the first buffer overflow are obtained, e.g., in [14,15], where the multiple vacation policy and an unreliable server subject to breakdowns are assumed, respectively, in the finite-buffer model with Poisson arrivals. As a tool for avoiding the risk of a buffer overflow, active queue management (AQM) is often applied. The representation for the queue-size distribution in a model with Poisson arrivals, AQM-type dropping function and finite buffer capacity is obtained, e.g., in [16].

In [17,18,19,20,21,22,23], wireless sensor networks are modeled applying queueing theory. In [24], the problem of congestion control and processing management is investigated using Markov chains. One can find a novel approach based on matrix-analytic method in studying discrete-time queues in [25]. In [26], the representation for the line-length distribution in the general-type discrete queue is obtained. Corresponding result for the case of a Batch Markovian Arrival Process (BMAP) in the discrete case can be found in [27]. Cost analysis for sensor network nodes accepting two different classes of packets is done in [28] basing on the finite-buffer Geo/G/1/K-type model with vacations. Distribution of queueing delay in discrete-time model is studied in [29].

As one can observe, most of analytical results relate mainly to performance measures of the stationary state of the system. However, transient (time-dependent) analysis is sometimes desired or even necessary, e.g., in the case of the observation of the system just after its restarting after a breakdown, or simultaneously with the implementation of a new control mechanism. In addition, in some practical situations, the queue-length behavior may be destabilized by different “outside” phenomena (like e.g., fade-out or interference occurring in wireless telecommunication).

In this paper, we study the model of a WSN node based on a finite-buffer discrete-time queueing system in transient state. The time axis is divided into fixed-length periods (called slots) and numbered by 1, 2, etc. Such an approach is usually used in modeling different real-life systems, for example in the analysis of telecommunication traffic. The in-depth analysis of various discrete-time queueing models can be found, e.g., in books [30,31,32]. Discrete-time queueing model with correlated arrivals and constant service times is analyzed in [33]. The case of generally distributed service times is investigated in [34]. In [35], discrete time queueing models and their networks are studied.

In this paper, a system of equations for the tail CDF of the first buffer overflow period duration conditioned by the initial state of the accumulating buffer is derived. The solution of the corresponding system written for probability generating functions (PGFs for short) is found using the analytical approach based on the idea of embedded Markov chain and linear algebra. Corresponding results for next buffer overflow periods are obtained as well. The numerical study illustrating theoretical results is attached. Therefore, the first main contribution of the paper is compact-form analytical results obtained for the transient state of the system, describing its evolution at arbitrary time (slot). Typically, in queueing modeling, the empty system is assumed initially. Thus, the next contribution of the paper is in showing an essential dependence (usually ignored) of the buffer overflow duration on the initial buffer state of the system. The dependence is visible in the formulae and it is illustrated via numerical results.

The remaining part of the article is organized as follows. In Section 2, we give a precise mathematical description of the considered queueing model and introduce some nomenclature. In Section 3, a transient system of equations for the tail CDF of the first buffer overflow period duration, conditioned by the number of packets accumulated in the buffer before the starting moment, is built. Moreover, a corresponding system written for PGFs is found there. Section 4 contains the main analytical result, namely a compact-form solution of the last system obtained in the previous section. The result for next buffer overflow periods is stated in Section 5. In Section 6, numerical analysis is provided. A brief summary and conclusion can be found in Section 7.

## 2. Model Description

In this paper, we consider a discrete-time queueing model in which the incoming packets arrive according to interarrival times being geometrically distributed with parameter 0<a<1 (a binomial arrival process), so the probability that an interarrival time equals *k* (time slots) is

(1) $$\begin{array}{cc} & a_{k}\overset{def}{=}a{\left(1-a\right)}^{k-1}, \end{array}$$

where k∈{1,2,...}.

The probability that an interarrival time exceeds *k* we denote by ${\bar{a}}_{k}.$ Obviously

(2) $$\begin{array}{c} {\bar{a}}_{k}=\sum_{i=k+1}^{\infty }a_{i}={\left(1-a\right)}^{k},\;k\geq 1. \end{array}$$

The *j*-fold convolution of the sequence $\left(a_{k}\right)$ with itself is defined as follows:

(3) $$\begin{array}{c} a_{k}^{1*}=a_{k},\;k\geq 1, \end{array}$$

and

(4) $$\begin{array}{c} a_{k}^{j*}=\sum_{i=1}^{k-1}a_{i}^{(j-1)*}a_{k-i}=\sum_{i=1}^{k-1}a_{i}a_{k-i}^{(j-1)*}, \end{array}$$

for 2≤j≤k.

Processing times are assumed to be of general distribution, where $b_{k}$ stands for the probability that the service time lasts *k* time units, where $\sum_{k=1}^{\infty }b_{k}=1.$ The maximum system capacity is assumed to be N, so we have an accumulating buffer with N−1 places and one place in service station. A natural processing discipline FIFO (First In First Out) is assumed.

In a single time slot, at most one job can arrive and one service can be finished. We accept the so-called arrival-first (AF) regime, at which if the arrival and departure appear at the same time (slot) an arrival takes precedence over a departure.

In this paper, we use the notation I{A} for the indicator (characteristic function) of the random event A.

## 3. Basic Equations for First Buffer Overflow Duration

In this section, we deal with the first buffer overflow duration $\gamma_{1}.$ We introduce the following notation for the conditional tail CDF of $\gamma_{1}:$

(5) $$\begin{array}{c} \Delta_{n}\left(k\right)\overset{def}{=}P\left\{\gamma_{1}\geq k\;|\;X_{0}=n\right\}, \end{array}$$

where k≥1,n∈{0,...,N−1} and $X_{0}$ stands for the number of packets present in the buffer just before the starting epoch.

Let us start with the case of the buffer being empty before the opening of the system (n=0). Note that the corresponding CDFs for n=0 and n=1 are equal, i.e.,

(6) $$\begin{array}{cc} P\{\gamma_{1}\geq k\;|\;X_{0}=0\} & =\sum_{r=1}^{\infty }a_{r}P\left\{\gamma_{1}\geq k\;|\;X_{0}=1\right\}=P\left\{\gamma_{1}\geq k\;|\;X_{0}=1\right\}, \end{array}$$

so $\Delta_{0}\left(k\right)=\Delta_{1}\left(k\right).$

Assume now that the system starts the operation with n∈{1,...,N−1} packets accumulated in the buffer. Let us denote by *i* (i≥1) the first departure epoch after the opening of the system at time 0. In addition, if i≥2, let *r* be the last arrival moment before i. As a result that departure epochs are Markov moments in the evolution of the considered system (due to memoryless property of geometric distribution of interarrival times), for fixed *n* the following random events are mutually exclusive:

- $\Lambda_{1}\left(n\right):$ the moment *r* is the arrival time of, at most, the (N−n−1)th packet and the next packet enters the system after time *i* (the buffer does not become saturated before time *i*);
- $\Lambda_{2}\left(n\right):$ the moment *r* is the arrival time of, at most, the (N−n−1)th packet and the next packet enters the system exactly at time *i*;
- $\Lambda_{3}\left(n\right):$ at time *r* the (N−n)th packet arrives, so the buffer overflow period begins at time r;
- $\Lambda_{4}\left(n\right):$ the first packet (after the opening of the system) arrives exactly at time *i*;
- $\Lambda_{5}\left(n\right):$ the first packet (after the opening of the system) arrives after time i.

Obviously, from the total probability law we get

(7) $$\begin{array}{c} \Delta_{n}\left(k\right)=\sum_{i=1}^{5}P\left\{\left(\gamma_{1}\geq k\right)\;\cap \Lambda_{i}\;|\;X_{0}=n\right\}. \end{array}$$

Let us note that the following representations are true:

(8) $$\begin{array}{c} P\left\{\left(\gamma_{1}\geq k\right)\;\cap \Lambda_{1}\;|\;X_{0}=n\right\}=\sum_{i=2}^{\infty }b_{i}\sum_{r=1}^{i-1}\sum_{j=1}^{N-n-1}a_{r}^{j*}{\bar{a}}_{i-r}\Delta_{n+j-1}\left(k\right), \end{array}$$

(9) $$\begin{array}{c} P\left\{\left(\gamma_{1}\geq k\right)\;\cap \Lambda_{2}\;|\;X_{0}=n\right\}=\sum_{i=2}^{\infty }b_{i}\sum_{r=1}^{i-1}\sum_{j=1}^{N-n-1}a_{r}^{j*}a_{i-r}\Delta_{n+j}\left(k\right), \end{array}$$

(10) $$\begin{array}{c} P\left\{\left(\gamma_{1}\geq k\right)\;\cap \Lambda_{3}\;|\;X_{0}=n\right\}=\sum_{i=2}^{\infty }b_{i}\sum_{r=1}^{i-1}a_{r}^{(N-n)*}I\left\{i-r\geq k\right\}, \end{array}$$

(11) $$\begin{array}{c} P\left\{\left(\gamma_{1}\geq k\right)\;\cap \Lambda_{4}\;|\;X_{0}=n\right\}=\Delta_{n}\left(k\right)\sum_{i=1}^{\infty }b_{i}a_{i} \end{array}$$

and

(12) $$\begin{array}{c} P\left\{\left(\gamma_{1}\geq k\right)\;\cap \Lambda_{5}\;|\;X_{0}=n\right\}=\Delta_{n-1}\left(k\right)\sum_{i=1}^{\infty }b_{i}{\bar{a}}_{i}. \end{array}$$

Observe that in the case of (9) (if j=N−n−1) and (11) (if n=N−1), according to AF regime, in fact, degenerated ”zero” buffer overflow periods occur at time *i* (the (N−n)th packet arrives and the service completes at this time).

Collecting the right sides of (8)–(12) and referring to (7), we obtain

(13) $$\begin{array}{cc} \Delta_{n}\left(k\right)= & \sum_{i=2}^{\infty }b_{i}\sum_{r=1}^{i-1}[\sum_{j=1}^{N-n-1}a_{r}^{j*}\left({\bar{a}}_{i-r}\Delta_{n+j-1}\left(k\right)+a_{i-r}\Delta_{n+j}\left(k\right)\right)\\ & +a_{r}^{(N-n)*}I\left\{i-r\geq k\right\}]+\sum_{i=1}^{\infty }b_{i}\left(a_{i}\Delta_{n}\left(k\right)+{\bar{a}}_{i}\Delta_{n-1}\left(k\right)\right), \end{array}$$

where n∈{1,...,N−1}.

Introduce now the following functionals:

(14) $$\begin{array}{cc} {\hat{b}}_{j}\overset{def}{=} & \sum_{i=2}^{\infty }b_{i}\sum_{r=1}^{i-1}a_{r}^{j*}a_{i-r}, \end{array}$$

(15) $$\begin{array}{cc} {\tilde{b}}_{j}\overset{def}{=} & \sum_{i=2}^{\infty }b_{i}\sum_{r=1}^{i-1}a_{r}^{j*}{\bar{a}}_{i-r}, \end{array}$$

(16) $$\begin{array}{cc} \hat{c}\overset{def}{=} & \sum_{i=1}^{\infty }b_{i}a_{i}, \end{array}$$

(17) $$\begin{array}{cc} \tilde{c}\overset{def}{=} & \sum_{i=1}^{\infty }b_{i}{\bar{a}}_{i} \end{array}$$

and

(18) $$\begin{array}{cc} \theta_{n}\left(z\right) & \overset{def}{=}\sum_{k=1}^{\infty }z^{k}\sum_{i=2}^{\infty }b_{i}\sum_{r=1}^{i-1}a_{r}^{(N-n)*}I\left\{i-r\geq k\right\}\\ & =\sum_{i=2}^{\infty }b_{i}\sum_{r=1}^{i-1}a_{r}^{(N-n)*}\sum_{k=1}^{i-r}z^{k}\\ & =\frac{z}{1-z}\sum_{i=2}^{\infty }b_{i}\sum_{r=1}^{i-1}\left(1-z^{i-r}\right)a_{r}^{(N-n)*}, \end{array}$$

where |z|<1.

If we define, moreover, the PGF of $\Delta_{n}\left(k\right)$ as follows:

(19) $$\begin{array}{cc} & {\hat{\Delta }}_{n}\left(z\right)\overset{def}{=}\sum_{k=1}^{\infty }z^{k}\Delta_{n}\left(k\right),\;\left|z\right|<1, \end{array}$$

then the system of Equation (13) can be rewritten in the following form:

(20) $$\begin{array}{cc} {\hat{\Delta }}_{n}\left(z\right)= & \sum_{j=1}^{N-n-1}\left({\tilde{b}}_{j}{\hat{\Delta }}_{n+j-1}\left(z\right)+{\hat{b}}_{j}{\hat{\Delta }}_{n+j}\left(z\right)\right)\\ & +\hat{c}{\hat{\Delta }}_{n}\left(z\right)+\tilde{c}{\hat{\Delta }}_{n-1}\left(z\right)+\theta_{n}\left(z\right), \end{array}$$

where n∈{1,...,N−1}. In addition (see (6)),

(21) $$\begin{array}{c} {\hat{\Delta }}_{0}\left(z\right)={\hat{\Delta }}_{1}\left(z\right). \end{array}$$

Putting

(22) $$\begin{array}{c} \tau_{j}\overset{def}{=}\left\{\begin{array}{cc} \tilde{c},\; & j=0,\\ {\tilde{b}}_{1}+\hat{c},\; & j=1,\\ {\tilde{b}}_{j}+{\hat{b}}_{j-1},\; & j\geq 2, \end{array}\right. \end{array}$$

the Equation (20) can be transformed as follows:

(23) $$\begin{array}{c} {\hat{\Delta }}_{n}\left(z\right)=\sum_{j=0}^{N-n-1}\tau_{j}{\hat{\Delta }}_{n+j-1}\left(z\right)+\theta_{n}\left(z\right), \end{array}$$

where n∈{1,...,N−1}.

## 4. Representation for Solution

In this section, we obtain an explicit solution of the system (21), (23) in a compact form. In [36], the idea of a potential of a random walk is considered. Namely, if the sequence $\left(Y_{n}\right)$ is defined as follows:

(24) $$\begin{array}{c} Y_{0}=0,\;Y_{n}=\sum_{k=1}^{n}X_{k}, \end{array}$$

where n≥1, and random variables $X_{1},X_{2},...$ are independent and identically distributed with $\tau_{k}\overset{def}{=}P\left(X_{n}=k\right),$ $k\geq 0,\tau_{0}>0,$ then a sequence $\left(R_{k}\right)$ defined in the following way:

(25) $$\begin{array}{c} \sum_{k=0}^{\infty }z^{k}R_{k}=\frac{1}{T(z)-1}, \end{array}$$

where

(26) $$\begin{array}{c} T\left(z\right)\overset{def}{=}\sum_{k=-1}^{\infty }z^{k}\tau_{k+1},\;\left|z\right|<1, \end{array}$$

is called the potential of the random walk $(Y_{n}).$ The representation (25) can be used to find successive terms of the potential $(R_{k}).$ Indeed, applying Maclaurin’s expansion, we can write

(27) $$\begin{array}{c} R_{k}=\frac{1}{k!}\frac{\partial }{\partial z}\left(\frac{1}{T(z)-1}\right){|}_{z=0}. \end{array}$$

However, from the other side, successive terms of $\left(R_{k}\right)$ can be found recursively, namely [36]

(28) $$\begin{array}{c} R_{0}=0,\;R_{1}=\frac{1}{\tau_{0}},\;R_{k}=R_{1}\left(R_{k-1}-\sum_{j=0}^{k-1}\tau_{j+1}R_{k-1-i}\right), \end{array}$$

where k≥2.

The potential has interesting algebraic applications. In [36], the following system of infinitely many linear equations is studied:

(29) $$\begin{array}{c} \sum_{j=-1}^{n-1}\tau_{j+1}{\hat{\delta }}_{n-j}-{\hat{\delta }}_{n}=\phi_{n},\;n\geq 1, \end{array}$$

where $\left({\hat{\delta }}_{n}\right)$ are unknowns and $\left(\tau_{n}\right)$ and $\left(\phi_{n}\right)$ are known sequences. It is proved that each solution of the system (29) can be represented in the following form:

(30) $$\begin{array}{c} {\hat{\delta }}_{n}=\beta R_{n}+\sum_{k=1}^{n}R_{n-k}\phi_{k},\;n\geq 1, \end{array}$$

where β is a certain constant and $\left(R_{k}\right)$ is the potential corresponding to the sequence $(\tau_{k}).$

As it turns out, the idea of the potential can be applied in solving the system (21), (23); however, firstly, it must be written in another, equivalent, form.

Introduce the following substitution:

(31) $$\begin{array}{c} {\hat{\delta }}_{n}\left(z\right)\overset{def}{=}{\hat{\Delta }}_{N-n}\left(z\right),\;n\in \left\{1,...,N\right\}. \end{array}$$

Observe that now the Equations (21), (23) can be rewritten as follows:

(32) $$\begin{array}{c} \sum_{j=-1}^{n-1}\tau_{j+1}{\hat{\delta }}_{n-j}\left(z\right)-{\hat{\delta }}_{n}\left(z\right)=\phi_{n}\left(z\right) \end{array}$$

for n∈{1,...,N−1}, and

(33) $$\begin{array}{c} {\hat{\delta }}_{N}\left(z\right)={\hat{\delta }}_{N-1}\left(z\right), \end{array}$$

where

(34) $$\begin{array}{c} \phi_{n}\left(z\right)\overset{def}{=}{\hat{\delta }}_{1}\left(z\right)\tau_{n}-\theta_{N-n}\left(z\right). \end{array}$$

Let us note that (32) has the form similar to (29); however, two essential differences can be observed. Firstly, the sequences of unknowns and free terms depend on the argument z. Secondly, the number of equations in the system (32) is finite in comparing to (29). In consequence, the representation (30) for the solution must be used in a slightly different form, namely

(35) $$\begin{array}{c} {\hat{\delta }}_{n}\left(z\right)=\beta \left(z\right)R_{n}+\sum_{k=1}^{n}R_{n-k}\phi_{k}\left(z\right),\;n\geq 1, \end{array}$$

where β(z) is certain function of variable *z* and $\left(R_{k}\right)$ is the potential corresponding to the sequence $\left(\tau_{k}\right)$ defined in (27) or (28). Next, the Equation (33) can be used for finding β(z) explicitly.

Let us start with substituting n=1 into (35). We get

(36) $$\begin{array}{c} \beta \left(z\right)=\frac{{\hat{\delta }}_{1}\left(z\right)}{R_{1}}={\hat{\delta }}_{1}\left(z\right)\tau_{0}. \end{array}$$

Next, substituting n=N and n=N−1 into (35) and, moreover, applying (33), we can easily eliminate ${\hat{\delta }}_{1}\left(z\right)$ as follows:

(37) $$\begin{array}{c} {\hat{\delta }}_{1}\left(z\right)=\frac{\sum_{k=1}^{N-1}\left(R_{N-k}-R_{N-1-k}\right)\theta_{N-k}\left(z\right)}{\sum_{k=0}^{N-1}\left(R_{N-k}-R_{N-1-k}\right)\tau_{k}}. \end{array}$$

Returning to ${\hat{\Delta }}_{n}\left(z\right)$ (by using the substitution (31)), we have

(38) $$\begin{array}{cc} {\hat{\Delta }}_{n}\left(z\right) & ={\hat{\delta }}_{N-n}\left(z\right)={\hat{\delta }}_{1}\left(z\right)\tau_{0}R_{N-n}+\sum_{k=1}^{N-n}R_{N-n-k}\phi_{k}\left(z\right)\\ & ={\hat{\delta }}_{1}\left(z\right)\tau_{0}R_{N-n}+\sum_{k=1}^{N-n}\left({\hat{\delta }}_{1}\left(z\right)\tau_{k}-\theta_{N-k}\left(z\right)\right)R_{N-n-k}\\ & ={\hat{\delta }}_{1}\left(z\right)\sum_{k=0}^{N-n}\tau_{k}R_{N-n-k}-\sum_{k=1}^{N-n}\theta_{N-k}\left(z\right)R_{N-n-k}. \end{array}$$

In consequence, referring to (33), (37) and (38), we can formulate the following main result:

**Theorem** **1.** *The PGF of the tail CDF of the first buffer overflow duration $\gamma_{1}$ in the considered queueing system can be represented as follows:*

(39) $$\begin{array}{cc} {\hat{\Delta }}_{n}\left(z\right)= & \frac{\sum_{k=1}^{N-1}\left(R_{N-k}-R_{N-1-k}\right)\theta_{N-k}\left(z\right)}{\sum_{k=0}^{N-1}\left(R_{N-k}-R_{N-1-k}\right)\tau_{k}}\sum_{k=0}^{N-n}\tau_{k}R_{N-n-k}-\sum_{k=1}^{N-n}\theta_{N-k}\left(z\right)R_{N-n-k}, \end{array}$$

*where n∈{0,...,N−1}, and the formulae for $\theta_{k}\left(z\right),$$\tau_{k}$ and $R_{k}$ are given in (18), (22) and (27) (or, equivalently, in (28)), respectively.*

## 5. The Case of Next Buffer Overflows

Let us denote by $\gamma_{r}$ the *r*th buffer overflow period duration (r≥2). Observe that the following representation is true:

(40) $$\begin{array}{c} P\left\{\gamma_{r}>k\right\}=P\left\{\gamma_{1}>k\;|\;X_{0}=N-1\right\}, \end{array}$$

where k≥1.

Indeed, the completion epoch of each buffer overflow period is a Markov moment in the evolution of the system. Therefore, the process of reaching each next buffer overflow period (beginning with the second one) is probabilistically identical as the one for the first period but with “initial” number of jobs accumulated in the buffer equal to N−1. In consequence, if we put

(41) $$\begin{array}{c} {\hat{\Delta }}^{⋆}\left(z\right)\overset{def}{=}\sum_{k=1}^{\infty }z^{k}P\left\{\gamma_{r}\geq k\right\}, \end{array}$$

where r≥2 and |z|<1, then we obtain the following:

**Remark** **1.** *The PGF ${\hat{\Delta }}^{⋆}\left(z\right)$ of the tail CDF of the rth buffer overflow duration $\gamma_{r}$ (r≥2) in the considered queueing system can be expressed as*

(42) $$\begin{array}{c} {\hat{\Delta }}^{⋆}\left(z\right)={\hat{\Delta }}_{N-1}\left(z\right), \end{array}$$

*where |z|<1 and the formula for ${\hat{\Delta }}_{N-1}\left(z\right)$ is given in (39).*

## 6. Numerical Study

In this section, we present the numerical study illustrating theoretical results. In particular, we are interested in the visualization of the impact on the distribution of the first buffer overflow duration for the following “input” parameters of the system:

- the offered traffic load ϱ defined as the quotient of the mean service time and the mean interarrival time;
- the number of jobs *n* accumulated in the buffer before the starting moment;
- the shape of the service (processing) time distribution;
- the buffer size.

In computations, we consider three types of the processing time distribution:

- geometric with fixed parameter b;
- deterministic (constant) of duration B=const;
- bounded discrete distribution, where the service time takes on finite number of possible values; dealing with the impact of the distribution skewness we analyze separately the following subcases of this type of distribution:
   **–** symmetric;
   **–** with positive skewness (positive asymmetry);
   **–** with negative skewness (negative asymmetry).

In [37], an algorithm of numerical inversion of probability generating function is proposed. Namely, if $F\left(z\right)=\sum_{k=0}^{\infty }f_{k}z^{k},$ where $|f_{k}|\leq 1$ and *z* ia a complex number, then $f_{k}$ for k=1,2,... can be approximated by ${\bar{f}}_{k}$ as follows:

(43) f¯k=12krk∑j=12k(−1)jRe(F(rexp(πij/k))),

where r∈(0,1). Moreover,

(44) $$\begin{array}{cc} & |f_{k}-{\bar{f}}_{k}|\leq \frac{r^{2k}}{1-r^{2k}}. \end{array}$$

We use the algorithm described above with r=0.1 to invert the right side of the Formula (39) in Theorem 1.

### 6.1. Impact of the Type of Processing Distribution

We investigate the impact of the type of processing distribution on the distribution of the first buffer overflow duration. Assume that a=0.25 so the mean interarrival time equals 4, and take N=11. Moreover, let us analyze three different processing time distributions with the same mean equal to 3 (so ϱ=0.75<1), namely geometric with parameter $b=\frac{1}{3}=0.333,$ deterministic with B=3 and bounded discrete distribution defined as

$$\begin{array}{c} b_{1}=b_{2}=b_{4}=b_{5}=\frac{1}{8},\;b_{3}=\frac{1}{2},\;b_{k}=0\;\mathrm{otherwise}. \end{array}$$

**Figure 1:**
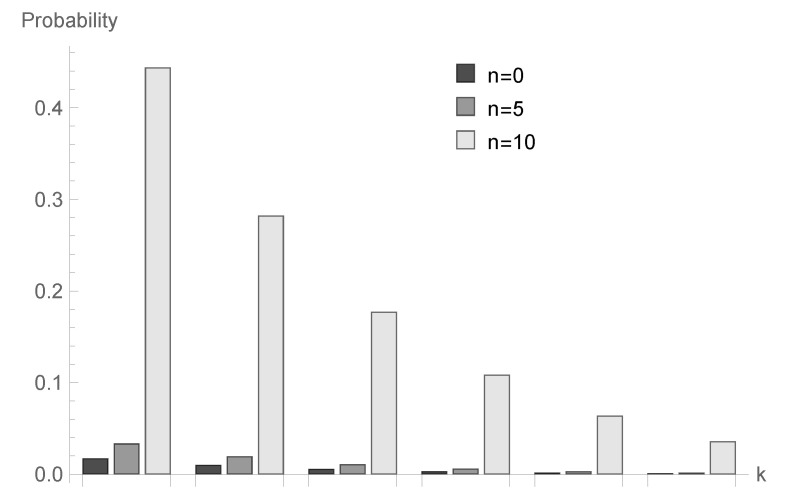
Conditional probabilities ${\hat{\Delta }}_{n}\left(k\right)$ for geometric processing distribution, ϱ=0.75 and n=0, 5 and 10.

**Figure 2:**
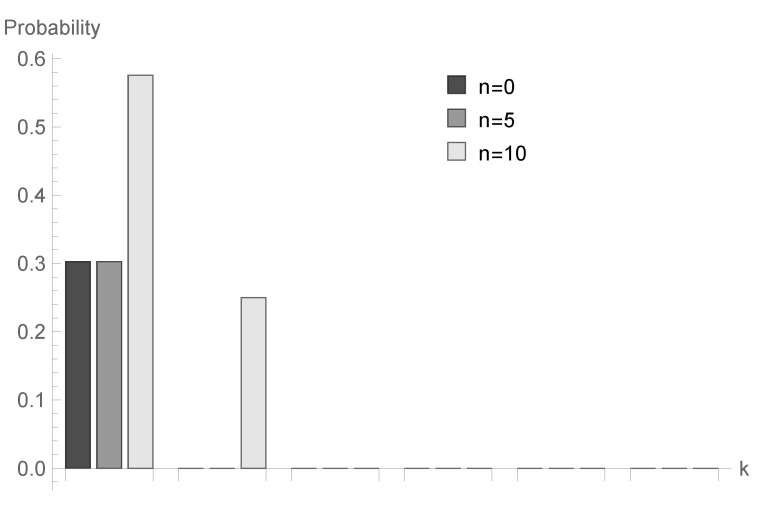
Conditional probabilities ${\hat{\Delta }}_{n}\left(k\right)$ for deterministic processing distribution, ϱ=1.00 and n=0,5 and 10.

**Figure 3:**
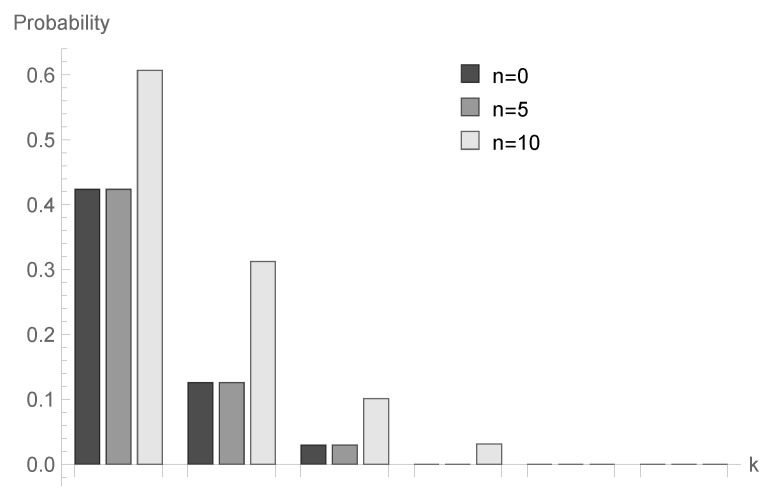
Conditional probabilities ${\hat{\Delta }}_{n}\left(k\right)$ for bounded discrete processing distribution, ϱ=1.25 and n=0,5 and 10.

In Figure 1, Figure 2 and Figure 3, conditional probabilities $\Delta_{n}\left(k\right)$ for k=1,2,...,6 are presented for n=0,5 and 10, respectively.

**Figure 4:**
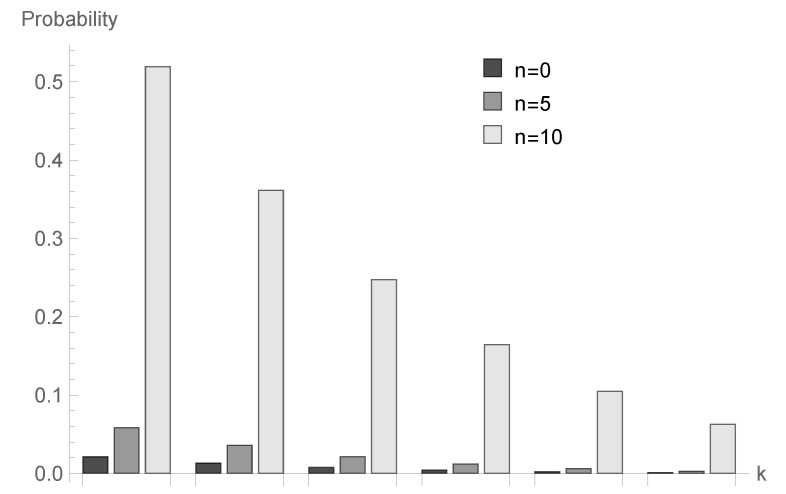
Conditional probabilities ${\hat{\Delta }}_{n}\left(k\right)$ for geometric processing distribution, ϱ=0.75 and n=0, 5 and 10.

**Figure 5:**
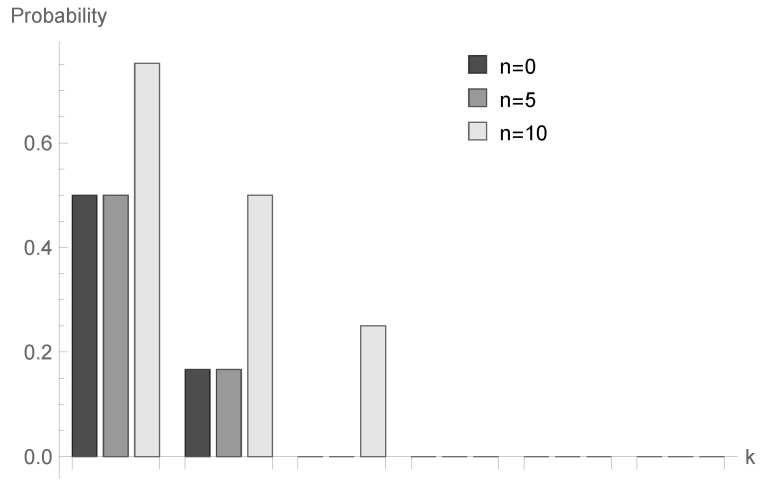
Conditional probabilities ${\hat{\Delta }}_{n}\left(k\right)$ for deterministic processing distribution, ϱ=1.00 and n=0,5 and 10.

**Figure 6:**
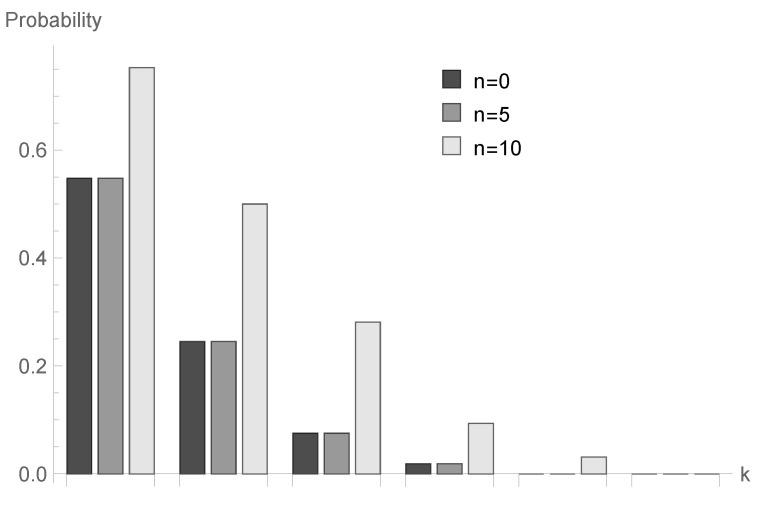
Conditional probabilities ${\hat{\Delta }}_{n}\left(k\right)$ for bounded discrete processing distribution, ϱ=1.25 and n=0,5 and 10.

In Figure 4, Figure 5 and Figure 6, the case of ϱ=1.00 is visualized. Assuming the same values of *a* and *N*, we present results for three processing time distributions with the same mean equal to 4: geometric with parameter $b=\frac{1}{4}=0.250,$ deterministic with B=4 and bounded discrete distribution defined as follows:

$$\begin{array}{c} b_{2}=b_{3}=b_{5}=b_{6}=\frac{1}{8},\;b_{4}=\frac{1}{2},\;b_{k}=0\;\mathrm{otherwise}. \end{array}$$

**Figure 7:**
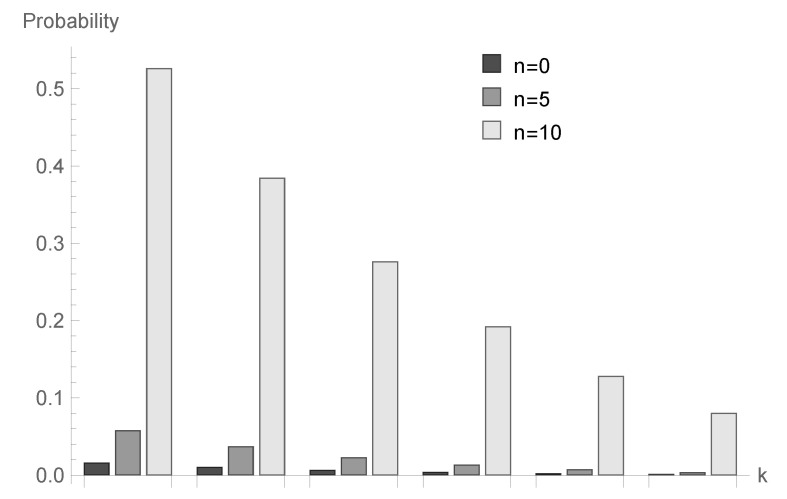
Conditional probabilities ${\hat{\Delta }}_{n}\left(k\right)$ for geometric processing distribution, ϱ=0.75 and n=0, 5 and 10.

**Figure 8:**
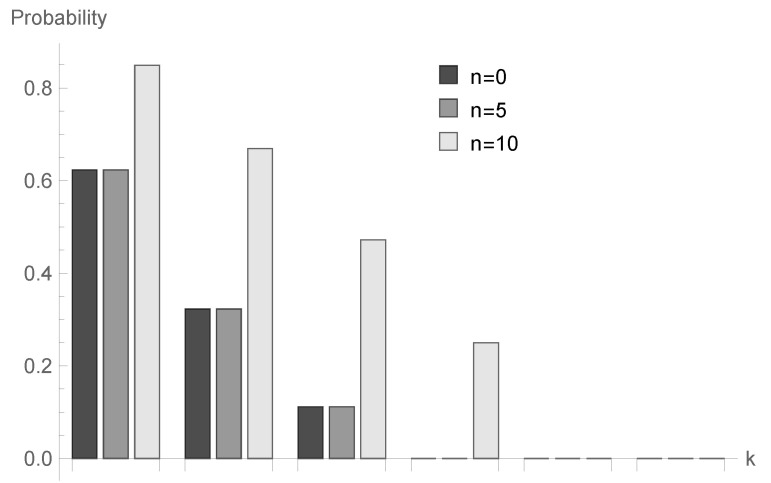
Conditional probabilities ${\hat{\Delta }}_{n}\left(k\right)$ for deterministic processing distribution, ϱ=1.00 and n=0,5 and 10.

**Figure 9:**
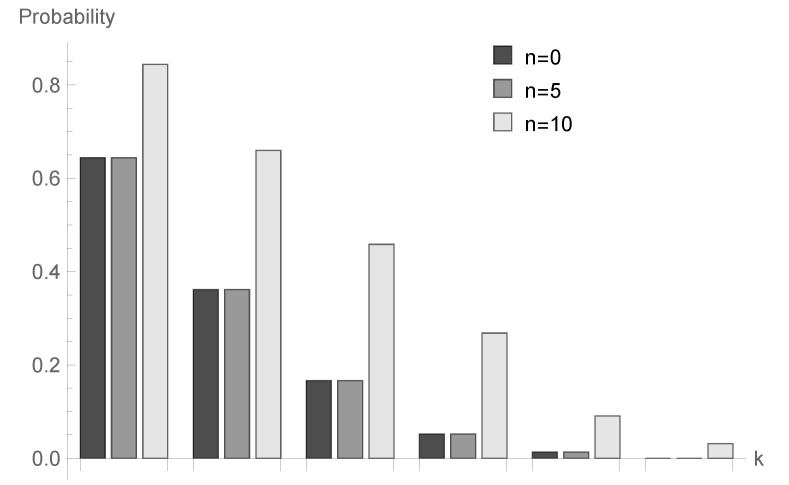
Conditional probabilities ${\hat{\Delta }}_{n}\left(k\right)$ for bounded discrete processing distribution, ϱ=1.25 and n=0,5 and 10.

Finally, the case of ϱ=1.25 and the mean service time equal to 5 are presented in Figure 7, Figure 8 and Figure 9. We take there geometric distribution with parameter $b=\frac{1}{5}=0.200,$ deterministic with B=5 and bounded discrete distribution given by

$$\begin{array}{c} b_{3}=b_{4}=b_{6}=b_{7}=\frac{1}{8},\;b_{5}=\frac{1}{2},\;b_{k}=0\;\mathrm{otherwise}. \end{array}$$

Evidently, as *k* increases, then the probability values

$$\begin{array}{c} {\hat{\Delta }}_{n}\left(k\right)=P\left\{\gamma_{1}\geq k\;|\;X_{0}=n\right\} \end{array}$$

for fixed *n* decrease; however, the shape of this relationship depends on the type of processing distribution. The relationship between the initial buffer state and the processing distribution type is interesting. Figure 1, Figure 4 and Figure 7 show that there is the biggest difference in the case of geometric distribution, where a huge disproportion between values obtained for n=0,5 and n=10 can be observed.

### 6.2. Impact of Skewness Type of the Processing Distribution

In this subsection, we investigate the effect of the statistical shape of the service type distribution on the tail of conditional distribution of the first buffer overflow duration in the considered model. Assume, as previously, that a=0.25,N=11, and accept a bounded discrete processing distribution. Consider three different types of this distribution in the case of ϱ=1 (so with the same mean), namely

- symmetric distribution of the form
  $$\begin{array}{c} b_{1}=b_{2}=b_{4}=b_{5}=\frac{1}{8},\;b_{3}=\frac{1}{2} \end{array}$$
  and $b_{k}=0$ otherwise, for which the skewness equals 0;
- distribution with positive skewness (positive asymmetry) of the form
  $$\begin{array}{c} b_{2}=\frac{7}{16},\;b_{3}=\frac{4}{16},\;b_{4}=\frac{3}{16},\;b_{5}=\frac{2}{16} \end{array}$$
  and $b_{k}=0$ otherwise, for which the skewness equals 0.629>0;
- distribution with negative skewness (negative asymmetry) of the form
  $$\begin{array}{c} b_{1}=\frac{2}{16},\;b_{2}=\frac{3}{16},\;b_{3}=\frac{4}{16},\;b_{4}=\frac{7}{16} \end{array}$$
  and $b_{k}=0$ otherwise, for which the skewness equals −0.629<0.

Let us note that means are the same and equal to 3.

**Figure 10:**
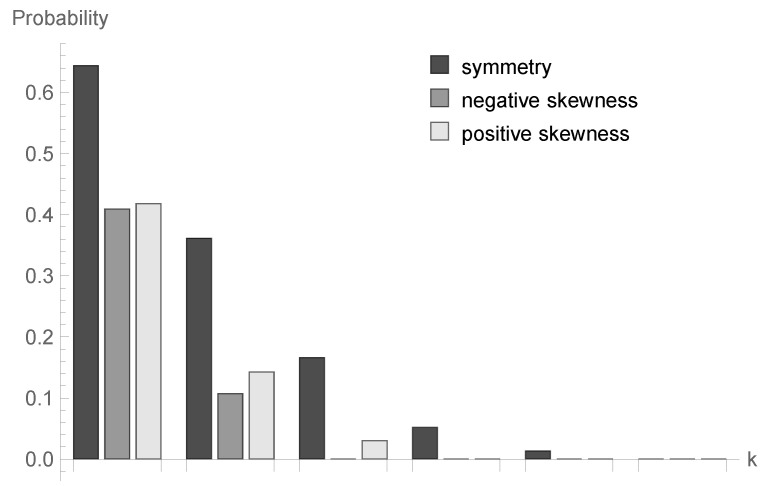
Impact of skewness type on conditional probabilities ${\hat{\Delta }}_{3}\left(k\right)$ for bounded discrete processing distribution and ϱ=1.

The values of probabilities ${\hat{\Delta }}_{3}\left(k\right)=P\left\{\gamma_{1}\geq k\;|\;X_{0}=3\right\}$ for k=1,...,6 are presented in Table 1 and in Figure 10. It is probably a bit surprising that these probabilities are the highest for a symmetric distribution. In the case of positive and negative skewness for *k*s greater than or equal to 3, the probabilities are very close to zero.

### 6.3. Mean Buffer Overflow Duration in Dependence on Offered Load and Initial Buffer State

Let us study now the impact of the offered load ϱ and the initial buffer state *n* on the mean first buffer overflow duration. Let us note that the mean conditional first buffer overflow duration $E_{n}\left(\gamma_{1}\right)$ can be obtained just from the formula (39), namely

$$\begin{array}{c} E_{n}\left(\gamma_{1}\right)=\lim_{z\to 1}{\hat{\Delta }}_{n}\left(z\right). \end{array}$$

**Figure 11:**
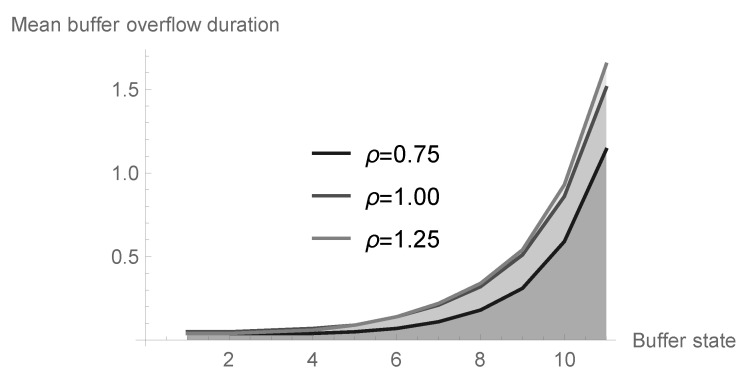
Mean first buffer overflow duration in dependence on offered load and initial buffer state.

Assume that N=11 and a=0.25, and analyze three different possibilities in the case of geometric-type processing distribution, namely b=0.33 (ϱ=0.75<1), b=0.25 (ϱ=1.00) and b=0.20 (ϱ=1.25>1). The results are presented in Table 2 and visualized in Figure 11. Note that the values increase with increasing offered load. For a small and medium level of initial buffer state, the differences are small. For greater values of *n* compared to *N*, the differences are significant. For example, the average duration of the first buffer overflow period for the value of n=10 increases almost twice as compared to the value obtained for n=9.

### 6.4. Impact of System Size

**Figure 12:**
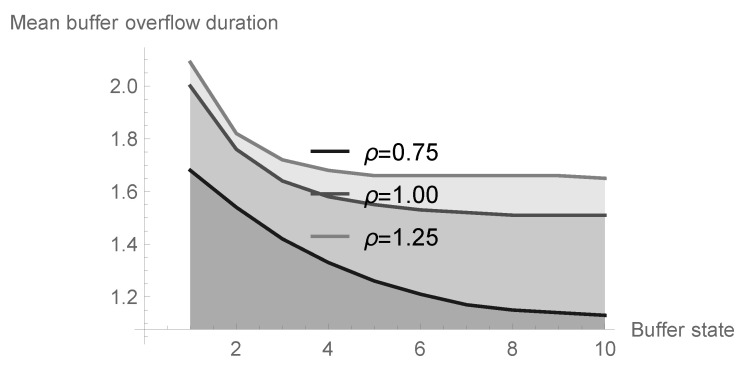
Impact of of system size on the mean first buffer overflow duration for different values of offered load.

Finally, in Table 3 and in Figure 12, the impact of the system size *N* on the duration of the first buffer overflow is illustrated for geometric processing distribution and system parameters kept the same as in the previous subsection. Here, n=N−1 is assumed; therefore, we can illustrate in this case not only the first buffer overflow but also next ones. Indeed, due to the fact that after finishing each buffer overflow period the number of accumulated packets equals N−1, this buffer state becomes an initial one for the second and next buffer overflow periods. The dependence on the offered load ϱ is similar to that analyzed in the previous case. The buffer overflow duration decreases with the increase of the declared buffer volume; however, the rate of this change is the most visible for small values of N. Generally, it shows that a large increase of the buffer capacity does not have an essential impact on the average number of lost packets, since mean buffer overflow durations do not differ significantly for large *N*s.

## 7. Conclusions

The possibility of probabilistic evaluation of the duration of the buffer overflow period is crucial in the evaluation of transmission quality and the network optimization process. The article proposes a probabilistic model for the functioning of a wireless sensor network node based on a queueing system with discrete time and a limited capacity of the buffer accumulating incoming data packets. Using the analytical approach based on the concept of the embedded Markov chain, the total probability formula and linear algebra, a compact representation for the PGF of the tail of the CDF of the first buffer overflow period is obtained, depending on the initial state of this buffer. As a simple conclusion, the appropriate formula is also found for the subsequent periods of buffer overflow. The numerical study examines the sensitivity of the buffer overflow time distribution on the packet arrival intensity, the type of service time distribution and the buffer filling level at the time of system start.

## Figures and Tables

**Table 1:** Impact of skewness type on conditional probabilities ${\hat{\Delta }}_{3}\left(k\right)$ for bounded discrete processing distribution and ϱ=1.

*k*	Symmetry	Negative Skewness	Positive Skewness
1	0.644089	0.409344	0.418129
2	0.361425	0.107254	0.142688
3	0.166121	4.163336 $\times \;10^{-14}$	0.030098
4	0.051831	6.938894 $\times \;10^{-14}$	0
5	0.013312	5.551115 $\times \;10^{-13}$	1.110223 $\times \;10^{-12}$
6	3.700743 $\times \;10^{-11}$	9.251859 $\times \;10^{-12}$	2.312965 $\times \;10^{-12}$

**Table 2:** Mean first buffer overflow duration in dependence on offered load and initial buffer state.

Buffer State *n*	ϱ=0.75	ϱ=1.00	ϱ=1.25
0	0.036321	0.049755	0.038306
1	0.036321	0.049755	0.038306
2	0.037954	0.055577	0.045982
3	0.042496	0.069353	0.062898
4	0.052282	0.094659	0.092465
5	0.071575	0.137233	0.140520
6	0.108269	0.206258	0.216532
7	0.177884	0.318161	0.337882
8	0.313364	0.508249	0.544020
9	0.585439	0.855341	0.925785
10	1.134407	1.511127	1.654884

**Table 3:** Impact of system size on the mean first buffer overflow duration for different values of offered load.

System Size *N*	ϱ=0.75	ϱ=1.00	ϱ=1.25
2	1.682303	2.000242	2.088127
3	1.535842	1.762685	1.818474
4	1.418714	1.639140	1.716005
5	1.327611	1.577548	1.678921
6	1.257000	1.545704	1.664732
7	1.205492	1.528632	1.658915
8	1.171707	1.519536	1.656483
9	1.151613	1.514796	1.655473
10	1.140419	1.512365	1.655056
11	1.134407	1.511127	1.654884
